# Research to action to address inequities: the experience of the Cape Town Equity Gauge

**DOI:** 10.1186/1475-9276-7-6

**Published:** 2008-02-04

**Authors:** Vera Scott, Ruth Stern, David Sanders, Gavin Reagon, Verona Mathews

**Affiliations:** 1 School of Public Health, University of Western Cape, South Africa

## Abstract

**Background:**

While the importance of promoting equity to achieve health is now recognised, the health gap continues to increase globally between and within countries. The description that follows looks at how the Cape Town Equity Gauge initiative, part of the Global Equity Gauge Alliance (GEGA) is endeavouring to tackle this problem.

We give an overview of the first phase of our research in which we did an initial assessment of health status and the socio-economic determinants of health across the subdistrict health structures of Cape Town. We then describe two projects from the second phase of our research in which we move from research to action. The first project, the Equity Tools for Managers Project, engages with health managers to develop two tools to address inequity: an Equity Measurement Tool which quantifies inequity in health service provision in financial terms, and a Equity Resource Allocation Tool which advocates for and guides action to rectify inequity in health service provision. The second project, the Water and Sanitation Project, engages with community structures and other sectors to address the problem of diarrhoea in one of the poorest areas in Cape Town through the establishment of a community forum and a pilot study into the acceptability of dry sanitation toilets.

**Methods:**

A participatory approach was adopted. Both quantitative and qualitative methods were used. The first phase, the collection of measurements across the health subdistricts of Cape Town, used quantitative secondary data to demonstrate the inequities. In the Equity Tools for Managers Project further quantitative work was done, supplemented by qualitative policy analysis to study the constraints to implementing equity. The Water and Sanitation Project was primarily qualitative, using in-depth interviews and focus group discussions. These were used to gain an understanding of the impact of the inequities, in this instance, inadequate sanitation provision.

**Results:**

The studies both demonstrate the value of adopting the GEGA approach of research to action, adopting three pillars of assessment and monitoring; advocacy; and community empowerment. In the Equity Tools for Managers Project study, the participation of managers meant that their support for implementation was increased, although the failure to include nurses and communities in the study was noted as a limitation. The development of a community Water and Sanitation Forum to support the Project had some notable successes, but also experienced some difficulties due to lack of capacity in both the community and the municipality.

**Conclusion:**

The two very different, but connected projects, demonstrate the value of adopting the GEGA approach, and the importance of involvement of all stakeholders at all stages. The studies also illustrate the potential of a research institution as informed 'outsiders', in influencing policy and practice.

## Background

### The challenge of using research to promote health equity

Equity is an ethical concept of social justice, fairness and human rights, where need rather than privilege is the foundation for the allocation of resources [1,2]. Whitehead [3] provides us with the helpful and much cited definition of health inequities being "differences in health that are unnecessary, avoidable, unfair and unjust". Concern about equity in health is not new. International health and development agencies, researchers and health practitioners have been highlighting the inequities between and within countries for decades [4]. Equity was one of the founding principles of the Primary Health Care approach, ratified in the Alma Ata Declaration of 1978 and a priority of the World Health Organisation (WHO) Health For All Strategy. Equity concerns are also prominent considerations in the 2000 Millennium Declaration, which gave rise to the Millennium Development Goals [4]. In South Africa, the importance of addressing equity is noted in key policy documents at a National, Provincial and Local Government level [5-7]. However, it is a principle that has proven difficult to realise, and growing inequities within and between countries have been clearly documented over the last two and a half decades [2,8]. The 1999 UNDP Human Development Report [9] demonstrates this clearly, showing how the income gap between the five richest countries in the world and the five poorest has shifted from 30 to 1 in 1960, to 74 to 1 in1997.

Over the past few years, a renewed interest in equity has led to a growing body of research, much of which focuses on identifying and describing the extent of inequities in health status and health service provision [10,1]. Less has been written on policy change to promote equity for action: only in the health care financing literature has this been addressed, and here work on resource allocation strategies predominates [11,12]. This has led to a call for additional research that investigates and informs public policy development [13], along with the use of existing data on inequities, shifting the emphasis of the research from analysis to action [14]. Included is the need for policies that will lift people out of poverty, given that it is unjust to allow them to live in poverty when adequate resources are available within society at large to rectify this [14].

### The Global Equity Gauge Alliance: a strategy to act on health inequity

The Global Equity Gauge Alliance (GEGA) was conceived in 2002 as part of this trend, as an active approach of *research to action *to bring about sustained reductions in inequities in health and health care. The approach involves 3 inter-related pillars: 1.) assessment and monitoring, 2.) advocacy and 3.) community empowerment. It has been successfully adopted by several GEGA initiatives [15]. The Cape Town Equity Gauge is part of this global movement.

### The context and approach of the Cape Town Equity Gauge

The Cape Town Metropole, like the rest of South Africa, has vast disparities between the wealthiest communities living in comfortable first world conditions, and the poorest, who live in conditions that are as bad as some of the worst found in developing countries [16]. These inequities owe much to the policies of the former apartheid regime, which included forced removals of Black people to peripheral, underserved areas; discriminatory job reservation for certain racial groups; and a legacy of poor education and training for Black communities, making it difficult for them to find employment or to afford housing, services or transport to and from low paying jobs. (Black in this instance is used to describe Africans, so called 'Coloureds' and Indians.) With the dismantling of Apartheid in the 1990s, newly established freedom of movement enabled large numbers of rurally based (overwhelmingly Black African) South Africans to migrate to the city from even greater conditions of poverty and deprivation, causing even more poverty and overcrowding in the city. This situation has been further aggravated by the increasing impact of HIV/AIDS, and macro-economic policies that have constrained social development [16]. It is within this context that the National, Provincial and Local Governments are struggling to meet the basic housing, infrastructure and services backlog in Cape Town.

Since health or ill-health is mainly determined by broad socio-economic and environmental factors such as income, housing, water and sanitation, rather than the availability of health services [17] it is not surprising that there are gross health inequities across Cape Town. Even the provision of health services is grossly inequitable with those who least require the services, having access to more varied and a greater proportion of them than those who require the services most [16].

The Cape Town Equity Gauge was established in 2002 as a response to these inequities as a collaboration between local and provincial governments, academic institutions, non-governmental and community based organisations, coordinated by the School of Public Health (SOPH) at the University of the Western Cape. The Cape Town Equity Gauge adopted a two-phased research to action strategy. The first phase was based on the assessment and monitoring pillar of the GEGA approach. It is the collection of measurements across the health subdistricts of Cape Town that demonstrate the inequities. The second phase consisted of a number of projects, identified as priorities by the Equity Gauge partners to address the inequities found in the first phase. These collectively build on all three pillars. In this paper we give an overview of the initial phase and then describe two projects from the second phase, which formed part of the larger body of equity research that we have done. The first project, the "Equity Tools for Managers Project" engaged health managers in the development of two tools to monitor and manage inequity in primary level health care provision (an Equity Measurement Tool and a Human Resource Allocation Tool). The second project, the "Water and Sanitation Project", engaged community structures and other sectors to address the problem of inadequate sanitation.

## Methods and Results

Research for action is best conducted through a participatory approach that enables ownership of the research by the researchers, the communities being researched and those who have the responsibility to implement the findings [18]. Participatory research also has the advantage of the research itself being used as a tool for change through the ongoing process of reflection and action [19,20]. A participatory approach has therefore been adopted by the Cape Town Equity Gauge. This means that the methods and results are inter-related, and so are presented together in this paper.

All the Cape Town Equity Gauge research was led by the School of Public Health, University of the Western Cape, in collaboration with other stakeholders. Both quantitative and qualitative methods were adopted. The first phase, the collection of measurements across the health subdistricts of Cape Town, used quantitative secondary data to demonstrate the inequities. In the Equity Tools for Managers Project further quantitative work was done, supplemented by qualitative policy analysis to study the constraints to implementing equity. The Water and Sanitation Project was primarily qualitative, using in-depth interviews and focus group discussions. These were used to gain an understanding of the impact of the inequities, in this instance, inadequate sanitation provision.

In the Equity Tools Project, informed consent was obtained from the two participating public sector institutions (City Health and Metro District Health Services) as well as the Provincial Health Department. Senior organizational managers participated in the conceptualization of the Cape Town Equity Gauge and formed part of the Cape Town Equity Gauge Management Task Team which commissioned, and which granted permission for the research within their own organisations. The Water and Sanitation Project, as a community based initiative, worked through the community structures to determine the nature and scope of the research, the issues to be covered in the interviews, and the identification of the sample, which included community members and key officials involved in the Project. Informed consent was given by all respondents and the results were taken back to the community for discussion before the final reports were written.

### Phase 1. Initial measurement of health inequities

The first research question posed by the participatory action research team was: Are there significant inequities in health across the health subdistricts of Cape Town?

A series of interactive workshops were held with the primary health care managers between March 2002 and March 2003. The health service partners (City Health and Metro District Health Service) nominated their subdistrict managers to participate in the process of identifying and measuring inequities. Most of the subdistrict managers (16 out of 22) participated actively and consistently in the workshop series. At the first workshop the researchers presented a theoretical understanding of equity as a concept of social justice based on balancing need and resources, and sought to reach consensus with the managers on this definition. From this point on, the role of the researchers became that of facilitators. Managers made decisions by reaching consensus. There were two meetings which were attended by all the subdistrict managers and included their organisational managers for the purposes of report back and further modification of the work.

Health managers were asked to list what they considered to be the various determinants of need for health services in the Cape Town context. They listed a range of indicators which were then grouped into three categories: demographic, health outcomes and underlying socioeconomic determinants. These were then looked at through an 'equity lens' – described by GEGA as moving beyond average measures to examine the differences between the various geographic and social groups. In this instance, the equity lens involved investigating the determinants by disaggregating secondary data to compare the 11 different geographical health subdistricts in Cape Town.

The secondary data used were taken from a wide range of sources, including the 1996 South African Census, health statistics collected by the City Health Department and HIV prevalence projections at the University of Cape Town. To be used, data sets had to be complete, reliable and valid. As can be seen from the graphs, this process provided a clear picture of the gross inequities in the underlying determinants of health [see Additional files 1, 2 and 3] and health outcomes [see Additional files 4, 5 and 6]. It also demonstrated a recurrent pattern of inequity with two health subdistricts, Subdistrict 5 and 7, consistently showing the greatest need for health services.

The initial measurement phase was instrumental in gaining increased support from the organisational and subdistrict managers to the overall Equity Gauge initiative, which set the scene for the collaborative approach that has been central to all areas of work.

### Phase 2, Project A. The Equity Tools for Managers Project: the process and the results

This first phase of measurement work convinced organisational and subdistrict managers that inequity was a significant problem in Cape Town. This prompted them to express a desire to address the inequity. This signified a second round in the participatory action research process which asked the following research question: Given the demonstrated inequities in health, does the primary level service allocate resources according to need? Again the managers set the agenda and the role of the researchers from the SOPH was to act as facilitators.

The organisational managers on the Cape Town Equity Gauge Management Task Team requested that the first area of equity research should focus on the primary level health services in Cape Town. Although they recognised that tackling the underlying socioeconomic determinants of health was essential, they felt that they should first address inequities within their own service provision (and this is their area of direct responsibility) before attempting to advocate for change within other sectors.

**Figure 1:**
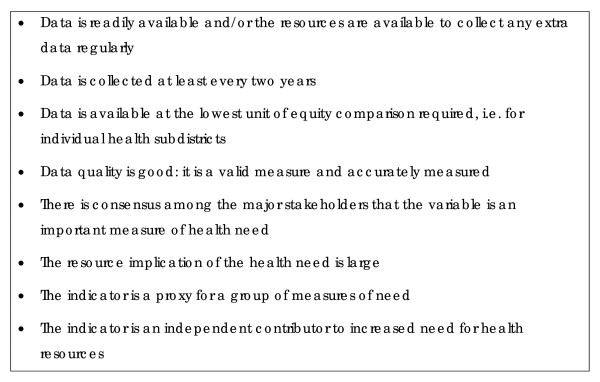
Selection criteria for indicators of health need in the Cape Town context. This is a list of criteria that health managers in Cape Town felt should be applied when selecting indicators of health need to be used in the Cape Town context.

The subdistrict managers were identified as key to implementation of equity actions, as they have direct responsibility for operationalising policy within the primary level services. They requested assistance in quantifying the inequity in a manner that would enable them to use the control they had over public primary health expenditure. The series of workshops continued. A technical support team was established with specialists invited to participate on the basis of their skills (in public health, health information systems, public sector financing, health policy and planning and health economics) and their familiarity with the Cape Town context. The role of the technical team was limited to specialist advice and the final decision-making power remained with the subdistrict managers. An *Equity Measurement Tool *was developed to quantify health need in each of the health subdistricts. The technical aspects of this tool are described in detail in a Cape Town Equity Gauge report [21]. Through the process of debate subdistrict managers set criteria for indicators of health need in the Cape Town context. These are shown in Figure 1. They also decided to weight the various indicators of need to create a composite measure of "need for primary level health services". This was then compared with public primary level health expenditure in each health subdistrict. The mismatch between the **need **for primary level health services and public primary level health **expenditure**, [as shown in Additional file 7], was dramatic, serving as a powerful source of advocacy. In the Additional file 7, the zero line represents equitable public primary level health expenditure. A bar above the line represents public primary level health expenditure in excess of what is equitable and a bar below the line indicates an expenditure deficit.

Once health managers understood the financial implications of addressing the health inequities between subdistricts, they raised their next concern which led to the third round of this participatory action research process. While national and provincial tiers of government are able to address inequity by changes in financial allocations to provinces and regions respectively, district health managers face a far more complex task. In health districts, significant changes in expenditure can effectively only be achieved by equitable reallocation of staff, as staff make up 70% of district expenditure. Faced with the magnitude of inequity in health expenditure and the difficulties in reallocating staff, the managers' commitment waned, and they identified a number of obstacles to implementing the equity strategy. At this point in the process, the role of SOPH researchers was questioned by some of the managers who felt under pressure to bring about equity change. They made it clear to the researchers that the role of implementation lay with management and that researchers had no part in this. The researchers agreed to this in principle. However, they argued that the difficulties in implementing equity did not justify denying social justice to communities living in Cape Town. Through a process of debate and boundary-setting, managers agreed that a tool to aid implementation would be helpful. The next research question was: How can equity in health service provision at primary level be achieved without disruption to efficient service delivery? This resulted in the development of an *Equity Resource Allocation Tool *to assist managers to plan staff allocation in a manner that is equitable. As primary level health services are currently under funded and there are many vacancies the tool was a useful source of advocacy to motivate for increased funding and as a guide to the allocation of new staff as they were appointed. Given the complexity and sensitivity of this task, the managers added various constraining factors (including efficiency, workload norms and the importance of not levelling down to the poorest standards) to equity-motivated staff shifts, which, they argued, were important given their other management mandates.

As part of the ongoing reflective learning process, the constraints were further explored. This was done using an in-depth qualitative health policy analysis study that looked at both the health managers' and the nurses' perceptions of the constraints faced in implementing equitable health care resource policies. Twelve in-depth interviews with organisational and subdistrict managers and 6 focus group discussions with facility-based nurses were done. The methods and results are described in detail in a separate article [22]. The findings showed that, while the legitimacy of equity as a policy goal was broadly accepted, resistance existed to the implementation strategy. In part this was due to role conflict: managers supported equity in terms of their strategic planning responsibilities for Cape Town, but also felt that that they had to secure maximum resources for their own subdistrict and knew that they could be unpopular as line managers with their frontline health workers if they were deployed to under-resourced subdistricts. Nurses, who felt that their main responsibility was to provide a high standard of client care, were also concerned that staff reductions in relatively over-resourced subdistricts would negatively affect the quality of client care offered.

Another key factor contributing to the resistance was a lack of workplace trust between staff and managers resulting from inadequate communication and poor consultation. Nurses were not involved in the decision-making process and they did not believe that the managers considered their well-being.

### Where are we now?

Despite these reservations, some equitable reallocation of nurses and environmental health practitioners has been undertaken by the City of Cape Town Health Department.

At the time of writing, an agreement has been reached to repeat the measurements, although this time the analysis would be done primarily by the Health Departments, with the SOPH providing a supporting role. This marks an important landmark in integrating the measurement into mainstream data collection, ensuring sustainability of the process.

### Phase 2, Project B. The Water and Sanitation Project: a process of community empowerment and the results

The second example of research to action is a project which was initiated because of the gross inequities in access to basic sanitation demonstrated in Phase 1. It was developed as a community-based initiative in two informal settlements (also referred to as shacks or slum dwellings) in the one of most disadvantaged subdistricts of Cape Town. Amongst the many disadvantages suffered by the residents, is a lack of sanitation within the informal settlements – a City of Cape Town report in late 2003 talked of an average of 105 people per toilet, generally 1 toilet per 7 households where toilets have been provided, with none in other areas. The toilets that did exist were shared 'bucket' toilets which are overused and/or vandalised. Furthermore, because of the high level of unemployment, most people are home during the day, which means these are often the only toilets they have access to. Accompanying the inadequate sanitation are high rates of worm infestation and diarrhoea, which was found by Groenewald et al [23] to be the third highest cause of mortality in under 5 year olds in that particular subdistrict in 2001, after HIV/AIDS and lower respiratory infections.

The Water and Sanitation Project developed a particularly strong emphasis on community empowerment, reflecting the evidence from many countries that, for water and sanitation programmes to be successful, there must be a demand for the facilities by the communities [24]. It grew out of a multisectoral initiative, established in response to a medical officer finding evidence of worm infestation among most of the children in the schools in the informal settlements (96% of 1000 children examined in 12 primary schools). Two main areas of work were focused on as a result of these findings. The first is a health promoting schools initiative, which involves a regular deworming programme, a curriculum development component, and the improvement of the water and sanitation facilities within the schools.

The second initiative, the focus of this paper, was the community based Water and Sanitation Project. This was initially established to target one of the determinants of diarrhoea – inadequate sanitation – through a pilot of dry sanitation toilets (toilets that are not connected to the sewage system). A participatory approach was adopted, working closely with the local communities as part of a multisectoral programme. This was led by a community-based Sanitation Task Team comprised of representatives from the informal settlement street committees, officials from the local government, and the Cape Town Equity Gauge/SOPH researcher. Site visits to see toilets in operation informed the decision about which toilets to pilot; the community representatives, through their street committees, decided on the twenty households that would test the toilets, using their local knowledge and judgement about candidates that they believed would remain committed to the Project; and Task Team members were involved in the installation of the toilets. The monitoring of the dry sanitation toilets was the responsibility of the City of Cape Town Health and Water Services Departments, and, apart from some minor maintenance problems which were easily remediable, they were found to work well. Assessing the acceptability of the toilets by the community was the responsibility of the Cape Town Equity Gauge. This was an important component of the study as there was initial resistance to the pilot as the sanitation provision was not the water-borne toilets that the community aspired to. A first set of in-depth interviews was held with representatives from the twenty households before the toilets were installed, for their perception on the current situation vis-à-vis the lack of sanitation in the informal settlements, and its impact on their health. A second set of interviews was undertaken after they had been using the toilets for between nine months and one year, for their view on the acceptability of the toilets and their feasibility for the informal settlements. Additional interviews with 10 key officials directly and indirectly involved in the pilot provided the professional perspective. The content analysis of the interviews showed that there was general acceptance of the toilets by the householders [25]. The main reason for their satisfaction was the fact that they did not have to share the toilets. This meant that the toilets were not overused, and the owners could keep them clean. The following quote sums up the views of the community respondents:

"It's unlike the first toilets whereby people were unable to enter and use them because of such things like the dirtiness. We enter these toilets as if you are entering the house ..."

The officials' comments supported the communities' assessment that dry sanitation could be a viable option for the informal settlements.

An important contributing factor was the role of the community members of the Task Team. As they were neighbours of the householders, they were trusted to have the interest of the community as their priority. They and the key officials had also spent a considerable amount of time with the householders explaining the technology and providing practical support.

The initial Task Team had been set up with a specific focus, and its membership of street committee representatives was appropriate for that focus. However, after some time, the structure was challenged for not being representative of the wider community of the subdistrict. This led to the establishment of a new, comprehensive community forum, the Water and Sanitation Forum, which has an extended remit to cover the whole district and all aspects of water and sanitation. This marked an important stage in the development of the Water and Sanitation Project, as it gained the recognition of the community establishment of the subdistrict. The Cape Town Equity Gauge took on the responsibility of building the capacity of the Forum members, to support them in their various roles. This included team building skills to work with (and at the same time challenge) public sector officials, and awareness-raising on issues of health and sanitation. Two focus group discussions, one with the Forum's Executive Committee and one with general members, were undertaken approximately two years after the Forum was established to assess its achievements and to note its limitations [26]. The strength of the Forum was seen as the commitment of members, and their desire to take the knowledge they had gained out into the wider community. However, two important concerns were noted. The first was what the Forum members described as limited commitment by the City of Cape Town, which was restricting their capacity to work for change.

*All of these things we talk about need support from the municipality....if the municipality could hear us then something might happen*.

The second concern, which was linked to the first, was the lack of funds to cover the expenses of the Forum. Funding had been allocated for community support, but due to bureaucratic procedures and differences in priorities between the community and the City of Cape Town, these funds did not materialise. This has been a significant problem for community members who are largely unemployed.

*The problems ...were coming to the meetings penniless hoping that you will get money [to cover transport] once you were there....then told that the money has not yet been received*.

### Where are we now?

The success of the initial dry sanitation pilot resulted in an extended pilot programme, this time led by the council. 70 more toilets were installed. However, these had shared use (four households per toilet), and the Forum members were not involved in the decisions about their distribution. This led to overuse of some units, which the technology could not sustain, while others remained unused due to unresolved tensions about who should have access to them. The second phase of the pilot was therefore considered to be unsuccessful, and dry sanitation has not been introduced into the area.

The Forum continued to meet despite the constraints. This was largely due to the commitment of its members, and sense of being able to make a contribution to their communities. As one Forum member noted

*... this has not dampened our spirit. When we look around and see people doing what we have taught them it's encouraging*.

This commitment was rewarded, as Forum members were invited to participate in the provincial and local government diarrhoea prevention programmes as the legitimate community representatives. In addition, the Cape Town Equity Gauge initiated a community action programme in 2006 which involved training Forum members to establish and run health clubs in the community. These initiatives were aimed at assisting the Forum members to achieve their objective of taking water and sanitation concerns into the community, as educators in their communities, and as advocates for improved water and sanitation provision. However, due to the limited capacity of the local and provincial governments to support the Forum and a lack of funding, its membership has dwindled, meetings are intermittent, and this has impacted on its ability to take forward the intended outreach programme. The remaining Forum members, however, remain committed and determined to continue the programme, despite these constraints.

## Discussion

### The value of the equity gauge approach

While the two projects we have described are very different, there is a synergy between them. Both are highlighting problems in the same communities, adopting the GEGA approach as a framework. The Equity Tools for Managers Project focused on the assessment and monitoring pillars, and included a strong element of advocacy within the approach. The Water and Sanitation Project, on the other hand, focused mainly on community empowerment, although also with a strong advocacy focus and an evidence base provided by the initial assessment and monitoring work. The two projects also share the same professional partners – the Local and Provincial Government departments – so that the GEGA approach is being reinforced and becoming integrated into the consciousness of these institutions.

### Key success factors in translating research to action

Where the research has influenced policy and practice we attribute this to two main factors: the involvement of relevant stakeholders, and the process of empowerment of stakeholders. The Equity Tools for Managers Project involved the public sector primary care health managers. They were identified as the key stakeholders as they had the authority and strategic responsibility to implement equity-promoting health resourcing strategies. Their involvement in defining and measuring parameters of health need in Cape Town served as a powerful advocacy exercise in placing health equity on the agenda. It also equipped them with information to challenge colleagues in other sectors, such as housing and sanitation, and their political bosses to address socioeconomic and service delivery inequities that affect the health of the people of Cape Town. The participatory approach facilitated their involvement and meant that they were able to influence the research agenda: they identified their own operational needs, in this instance management tools to enable them to address health service delivery inequity.

The participatory research process was not always easy, as the Project descriptions demonstrate. While the managers initially welcomed the development of the Equity Measurement Tool, they began to feel threatened by the implications of having to allocate staff according to equity criteria, rather than the traditional historical and workload factors. This impacted on the relationship with the researchers and a careful negotiation process ensued which was ultimately beneficial to both sides: the mandate of the researchers was further extended to support managers in the development of a second tool (the Resource Allocation Tool). This tool empowers managers to act on health service delivery inequity within their managerial constraints. Being part of the process of tool development (as opposed to being passive recipients of a tool as an end product) has further increased managers' understanding of health equity and strengthened their capacity to manage their services equitably. This process supports the reflections of Braveman [13] who notes that achieving equity involves '*swimming against the tide*' of prevailing forces. Information, she argues, therefore needs to be placed within the wider context of strategic development that is cognisant of the forces and key actors.

The Water and Sanitation Project again illustrates the potential of involving and empowering stakeholders. The significant achievement in this project was the growth of a strong community Forum as a lobby for improved water and sanitation, through the exploration of context-appropriate technology and the intensive capacity building programme. The initial acceptance of the dry sanitation toilets owed much to the involvement of the community through the initial Task Team, and the decision to extend the pilot by the municipality was evidence of the success of the pilot. Setting up the revised Water and Sanitation Forum, while contentious at the time, led to support by the community power-base, which opened the door to networks and stronger sources of advocacy. The existence of the Forum provided the public sector with a legitimate entry point into the community, as shown by the involvement of Forum in the Health Department's diarrhoea prevention programmes. However, the failure of the second phase of the dry sanitation pilot and the difficulty in sustaining the Forum has demonstrated the tensions between supporting community based initiatives and responding to other pressing priorities within the community. This is particularly the case where problems are immense and multiple, and the capacity to respond to these are limited.

### Limitations in our experience

The two case studies also illustrate some of the difficulties of turning the concept of equity into action. In particular, the projects experienced the constraints of working with government departments, resulting from the constraints faced by these departments. These constraints were not unexpected. Achieving pro-equity change requires deep, long term commitment and a willingness to make changes to achieve health, which Gwatkin [14] points out is one of the leading challenges faced by those concerned with addressing health inequities. Part of this challenge is the need for flexibility and willingness to change on the part of large public sector bureaucracies that are, by their very nature, inflexible and resistant to change [27,28]. It also requires a willingness by individuals to make what could be difficult changes when they are often already working in stressful environments. Inevitably there is the difficult challenge noted by Whitehead and Dahlgren [29] to reduce inequities by improving health and service provision by levelling up, not down. Our work has probed, and sought solutions to some of the constraints managers face in Cape Town.

Fortunately, because we adopted a participatory process, we were able to reflect on our practice, and this meant that we were able to address some of these oversights. In the case of Equity Tools for Managers Project we worked with managers as the key stakeholders, given their role and authority to implement an equitable resource policy. In this we neglected the nurses who, as the backbone of the primary care service, would be the staff cadre most affected by a new resource allocation policy. Not being involved during the early stages of the measurement, analysis and the decision-making process, resulted in resentment and resistance among nurses. Nurses are powerful "street level bureaucrats" [30] and are able to derail the implementation of policy. This is well described in the literature by those who see policy implementation as an interactive process [31] where implementers influence policy change through their interpretation of policy goals and the decisions that they take in implementation:

We also found that managers were reluctant to use the Resource Allocation Tool because they anticipated resistance from the nurses. The work in the Equity Tools for Managers Project would also have been much stronger had it involved community structures from the start, as the community would have been powerful advocates in working with managers. This benefit has been described by Sanders et al [32], who note that research findings are more successfully implemented when they are part of campaigns that mobilise communities. Earlier involvement of communities would also have served to build strategic relationships between them and the managers. Finally, our participatory approach working with health managers did mean that the first of our key projects focused on addressing inequity in health service delivery, whereas more equity in health might have been achieved if inequity in the underlying socioeconomic factors had been addressed [17].

The Water and Sanitation Project, by contrast, involved the community from the start. Here the limitation was the resistance of some of the public sector officials to embrace the community participation approach, fuelled in part by their limited capacity. While they were committed in theory, this did not translate sufficiently into action. Pressure to provide additional sanitation to a drastically under-resourced area has meant that decisions were taken by the City of Cape Town to implement shared toilets without community involvement, a pragmatic response to an overwhelming problem, but one which has, in the end, been self limiting. The failure to sustain the success of the pilot illustrates the importance of demand led sanitation [24] noted earlier.

The mismatch in priorities between the council and the community which led to funds not being allocated to sustain the Forum clearly had a detrimental effect, both in terms of the funding itself, and the lack of trust that resulted. The substantial ground work undertaken to build the Forum, the commitment of its members, and the opportunities to become involved in new activities sustained it for a while, but ultimately, the pressures on both the community and the local government became too great. Once again, there are lessons to be learnt from the study. In this case, the focus of the Equity Gauge in gaining the commitment of senior managers was not strong enough. While attempts were made to involve them from the start, this had been difficult due to their other commitments, and so the emphasis shifted to advocacy in support of the communities, rather than full involvement of the managers in the process. A stronger emphasis on managerial participation, with the view to them being an integral part of the programme, would have facilitated greater mutual understanding, including the need and means to work collaboratively for long term gain, despite short term inconvenience.

## Conclusion

As noted, the experience of achieving equity is complex the world over and, so far, has had limited success. However, we are currently operating in a climate that is recognising and endeavouring to address inequities in health. GEGA in general, and the Cape Town Equity Gauge in particular, see themselves as part of this movement, acting as catalysts for change through an active approach that promotes pro-equity policies and social change.

This paper has outlined the experiences of two very different, but connected, projects, and the need to integrate all relevant stakeholders in approaches to achieve equity from the outset. In our endeavour to achieve the above, we have adopted a participatory approach, so that we would be able to benefit from our reflections, and involve the relevant stakeholders in seeking the solutions. We have also highlighted the many barriers we faced and lessons we learnt through this journey.

The difficulties that arise in using research for action highlights the wisdom of the GEGA approach which is that the three pillars overlap and strengthen each other, that they should all be an integral part of the process, and that they should be ongoing, rather than once-off activities. Assessment and monitoring not only monitors changes in the current state of health equity. There is also a need to monitor the implementation of community and health service plans to address inequity. Community empowerment provides a valuable source of advocacy, and community perspectives are valuable in assessment and monitoring. At the same time, advocacy raises the profile of the issues and the solutions, adding weight to the arguments and strengthening the communities in the process. In our experience, however, commitment to equity goals and the development of strategies to address inequity have not automatically translated into action. In both case studies, unexpected constraints arose which had the potential to halt the equity initiatives. Research was required to identify some of these constraints with the view to facilitating a process to address them in partnership with the community and health authorities.

A positive lesson for us all has been the value of a research institution, such as the School of Public Health, in programmes that aim to promote change. As outsiders to the decision-making bodies, but outsiders with powerful information, we have a valuable role to play, providing evidence upon which decisions can be made, and to use that to lobby for change that may be resisted by the policy makers and managers. This role has been highlighted by Baum [33] in her argument that researchers involved in social and political change should move beyond being 'objective' technicians that leave the business of policy and practice to others. Rather, she argues, we have the responsibility to use our research to shift agendas. For, as Baum points out, there is little point in conducting research if it does not leave the world in a different, and hopefully better, place.

## Competing interests

The author(s) declare that they have no competing interests.

## Authors' contributions

The main authors of the paper are VS and RS. They jointly wrote the abstract, background, methodology, discussion and conclusion.

VS wrote Phase 1 the section on Project A and the constraints to equity, work that she did jointly with GR and VM respectively. RS wrote Project B the section on the Water and Sanitation Project, which was work that she was instrumental in developing.

DS, the head of our department, was involved in conceiving the overall Cape Town Equity Gauge initiative, has been involved in both areas of work in a conceptual and advisory capacity. All authors read and approved the final manuscript.

## Supplementary Material

Additional file 1 Graph 1. % Households in Subdistricts of Cape Town living in informal dwellings, 1996. This is a bar graph which shows the percentage of households in each health subdistrict of Cape Town which lives in informal dwellings in 1996. Click here for file

Additional file 2 Graph 2. % Households in Subdistricts of Cape Town below poverty line. This is a bar graph which shows the percentage of households in each health subdistrict of Cape Town which lives below the poverty line in 1996. Click here for file

Additional file 3 Graph 3. % Households in Subdistricts of Cape Town without piped water in dwelling or on site, 1996. This is a bar graph which shows the percentage of households in each health subdistrict of Cape Town which do not have piped water in their dwelling or on site in 1996. Click here for file

Additional file 4 Graph 4. Infant Mortality Rate in Subdistricts of Cape Town, 2001. This is a bar graph which shows the number of infant deaths per 1000 live births in each health subdistrict of Cape Town in 2001. Click here for file

Additional file 5 Graph 5. HIV prevalence in general population in Subdistricts of Cape Town, 2001. This is a bar graph which shows the HIV prevalence in the general population in each health subdistrict of Cape Town in 2001. Click here for file

Additional file 6 Graph 6. Premature mortality in Subdistricts of Cape Town, measured in years of life lost (YLL), 2001. This is a bar graph which shows premature mortality in each health subdistrict of Cape Town as measured by years of life lost in 2001. Click here for file

Additional file 7 Graph 7. Public Primary Health Expenditure in Subdistricts of Cape Town, expressed in South African Rand, in Excess/Deficit of Equity. This is a bar graph which shows the public primary care expenditure in each health subdistrict of Cape Town in excess (a bar above the horizontal axis) or deficit of what is equitable (a bar below the horizontal axis) in 2001. Click here for file

## References

[B1] Braveman P, Gruskin S (2003). Defining equity in health. Journal of Epidemiology and Community health.

[B2] Braveman P, Tarimo E (2002). Social inequalities in health within countries: not only an issue for affluent nations. Social Science & Medicine.

[B3] Whitehead M, Townsend P, Davidson (1990). The Health Divide. Inequalities in Health.

[B4] WHO Task Force on Research Priorities for Equity in Health. & the WHO Equity Team (2005). Priorities for research to take forward the health equity policy agenda. Bulletin of the World Health Organisation.

[B5] South African Department of Health National Health Act 2004. Government Gazette, Cape Town.

[B6] Health Western Cape (2003). Healthcare 2010: Health Western Cape's plan for ensuring equal access to quality health care.

[B7] City of Cape Town Reporting on our achievements.

[B8] Evans T, Whitehead M, Evans T, Whitehead M, Diderichsen F, Bhuiya A, Wirth M (2001). Developing the Policy Response to Inequities in Health: A Global Perspective. Challenging Inequities in Health: From Ethics to Action.

[B9] United Nations Development Programme (1999). Human Development Report 1999.

[B10] Gilson L, Doherty J, Lake S, McIntyre D, Mwikisa C, Thomas S (2003). The SAZA study: implementing health financing reform in South Africa and Zambia. Health Policy and Planning.

[B11] McIntyre D, Muirhead D, Gilson L (2002). Geographic patterns of deprivation in South Africa: informing health equity analyses and public resource allocation strategies. Health Policy and Planning.

[B12] Bossert T, Chitah M, Bowser D (2003). Decentralisation in Zambia: resource allocation and district performance. Health Policy and Planning.

[B13] Braveman P (2003). Monitoring equity in health and health care: a conceptual framework. Journal of Popul Nutrition.

[B14] Gwatkin DR (2000). Health Inequality and the Health of the Poor: What Do We Know? What Can We Do?. Bulletin of the WHO.

[B15] McCoy D, Bambas L, Acurio D, Baya B, Bhuiya A, Chowdhury AM, Grisurapong S, Yualnli L, Ngom P, Ngulube TJ, Ntuli A, Sanders D, Vega J, Shukla A, Braveman P (2003). Global Equity Gauge Alliance: Reflections on Early Experiences. J Health Popul Nutr.

[B16] Sanders D, Chopra M (2006). Key challenges to achieving Health for All in an inequitable society: the case of South Africa. American Journal of Public Health.

[B17] Irwin A, Valentine N, Brown C, Loewenson R, Solar O, Brown H, Koller T, Vega J (2006). The Commission on Social Determinants of Health: Tackling the Social Roots of Health Inequities. PLoS Med.

[B18] Springett J, Leavey C, Bruce N (1995). Participatory Action Research: the development of a paradigm, dilemmas and prospects. Research and Change in Urban Community Health.

[B19] Bless C, Higson-Smith C (2000). Fundamentals of Social Research Methods – An African Perspective.

[B20] Laws S, Harper C, Marcus R (2003). Research for Development, a practical guide.

[B21] Scott V, Reagon G (2005). The Equity Measurement Tool Report to Funder.

[B22] Scott V, Mathews V (2005). Constraints faced by actors to achieving equity Report to Funder.

[B23] Groenewald P, Bradshaw D, Nojilana B, Bourne D, Nixon J, Mahomed H, Daniels J (2003). Cape Town Mortality, 2001 Part 1: Cause of death premature mortality.

[B24] Cairncross S (1992). Sanitation and water supply: practical lessons from the decade. Water and Sanitation discussion paper no 9.

[B25] Stern R, Mokgatle MJ, Mayosi B (2004). The Acceptability of Dry Sanitation: a preliminary study in two informal settlements in Khayelitsha, Cape Town.

[B26] Stern R, Zulu J, Tsolekile L, Mayosi B, Dayille N (2005). The role and progress of the Khayelitsha Water and Sanitation Forum, and opportunities for future development, Report of an evaluation.

[B27] Pettigrew A, Fenton E (2000). The Innovating Organisation.

[B28] Stern R, Green J (2005). Boundary workers and the management of frustration: a case study of two Healthy City partnerships. Health Promotion International.

[B29] Whitehead M, Dahlgren G Levelling up (part 1) A discussion paper on concepts and principles for tackling social determinants in health.

[B30] Walker L, Gilson L (2002). We are bitter but we are satisfied: Nurses as street-level bureaucrats in South Africa. Social Science and Medicine.

[B31] Walt G (1994). Health Policy, an introduction to process and power.

[B32] Sanders D, Labonte R, Baum F, Chopra M (2004). Making research matter: a civil society perspective on health research. Bulletin of the World Health Organization.

[B33] Baum F, Bruce N (2003). Research and Policy to Promote Health: What is the relationship?. Research and Change in Urban Community Health.

