# Supplementary material for: Research to action to address inequities: the experience of the Cape Town Equity Gauge
Source: Int J Equity Health. 2008 Feb 4;7:6. doi: 10.1186/1475-9276-7-6 (PMC2275279; doi:10.1186/1475-9276-7-6)
Supplement: Additional file 1 — Graph 1. % Households in Subdistricts of Cape Town living in informal dwellings, 1996. This is a bar graph which shows the percentage of households in each health subdistrict of Cape Town which lives in informal dwellings in 1996. [file 1475-9276-7-6-S1.doc]

# Research to action to address inequities: the experience of the Cape Town equity gauge

## Showing inequities and the mismatch of resource allocation
